# Thermal Management in Multi-Stage Hot Forging: Computational Advances in Contact and Spray-Cooling Modelling

**DOI:** 10.3390/ma18143318

**Published:** 2025-07-15

**Authors:** Gonzalo Veiga-Piñeiro, Elena Martin-Ortega, Salvador Pérez-Betanzos

**Affiliations:** 1 Instituto de Física e Ciencias Aeroespaciais (IFCAE), Universidade de Vigo, Campus As Lagoas, 32004 Ourense, Spain; 2 CIE—Galfor Company, P.I. San Cibrao das Viñas, Calle 2, 3, 32901 Ourense, Spain

**Keywords:** forging processes, thermal simulation, contact heat transfer, spray-cooling model, numerical modelling

## Abstract

Innovative approaches in hot forging, such as the use of floating dies, which aim to minimise burr formation by controlling material flow, require precise management of die geometry distortions. These distortions, primarily caused by thermal gradients, must be tightly controlled to prevent malfunctions during production. This study introduces a comprehensive thermal analysis framework that captures the complete forging cycle—from billet transfer and die closure to forging, spray-cooling, and lubrication. Two advanced heat transfer models were developed: a pressure- and lubrication-dependent contact heat transfer model and a spray-cooling model that simulates fluid dispersion over die surfaces. These models were implemented within the finite element software FORGE-NxT to evaluate the thermal behaviour of dies under realistic operating conditions. These two new models, contact and spray-cooling, implemented within a full-cycle thermal simulation and validated with industrial thermal imaging data, represent a novel contribution. The simulation results showed an average temperature deviation of just 5.8%, demonstrating the predictive reliability of this approach. This validated framework enables accurate estimation of thermal fields in the dies, and offers a practical tool for optimising process parameters, reducing burr formation, and extending die life. Moreover, its structure and methodology can be adapted to various hot forging applications where thermal control is critical to ensuring part quality and process efficiency.

## 1. Introduction

Hot forging is a fundamental manufacturing process used to produce high-performance components by deforming metals at elevated temperatures under significant pressure. It is widely employed in the automotive, aerospace, railway, and energy sectors, due to its ability to deliver excellent mechanical properties, material efficiency, and dimensional precision [1,2]. Typical applications include crankshafts, turbine blades, gears, and structural brackets. By enabling complex geometries and reducing internal defects [3,4], hot forging supports the production of critical parts under demanding quality standards. However, the extreme thermal and mechanical loads involved in the process present major technical challenges, especially for the tooling system. In particular, forging dies are exposed to repeated thermal cycling and intense contact with hot materials, which can lead to thermal fatigue, plastic deformation, and premature failure if not properly controlled.

In this context, the thermal behaviour of forging dies becomes a critical factor influencing the quality, performance, and cost-effectiveness of forged components. Uncontrolled or excessive die temperatures can lead to detrimental effects, such as thermal fatigue cracks, plastic deformation, and premature tool failure, ultimately compromising component integrity and process reliability [5,6,7]. Consequently, effective thermal management is paramount, especially in advanced forging systems incorporating elements like floating dies. Precise control not only extends die lifespan but also ensures the production of defect-free parts and prevents operational malfunctions. Recognising this importance, significant research efforts have focused on accurately modelling and predicting the thermal field within forging dies during operation [8,9,10].

Considerable research has utilised numerical techniques, primarily the Finite Element Method (FEM), to simulate and understand the complex thermal dynamics in different industrial processes [11,12,13], particularly in forging dies. Studies have investigated temperature distributions under various process conditions [14,15], analysed the influence of die temperature on material flow, and explored strategies like optimised die cooling to enhance service life by mitigating wear and thermal softening [16]. Furthermore, coupled thermomechanical damage analyses have provided valuable insights into die failure mechanisms under combined thermal and mechanical loads [17]. These physics-based modelling approaches have substantially advanced the understanding of heat transfer phenomena in forging tools.

Complementing traditional physics-based modelling, data-driven approaches using Artificial Intelligence (AI) and Machine Learning (ML) are emerging as powerful tools for thermal analysis and control in manufacturing. Techniques like neural networks, Gaussian processes, or surrogate modelling are being explored to predict temperature evolution rapidly, optimise cooling strategies, or perform real-time process monitoring based on sensor data or simulation results [18,19].

Despite the progress achieved through both FEM and emerging AI techniques, challenges persist. Many existing physics-based models are developed for specific forging scenarios, potentially limiting their generalisation. Moreover, accurately capturing the intricate physics of transient thermal contact between the die and billet, as well as the effectiveness of die cooling mechanisms (like spraying), remains a critical hurdle for achieving high predictive accuracy across diverse operational conditions.

This work addresses the existing limitations by presenting a comprehensive and experimentally validated methodology for modelling the thermal behaviour of forging dies. We introduce an enhanced thermal model that incorporates refined sub-models for die–billet contact heat transfer and spray-cooling efficiency, providing a robust and adaptable framework that improves the accuracy of die temperature predictions. This, in turn, enables better process control and extends tool life. The accuracy of the proposed numerical model was rigorously validated against experimental thermal imaging data obtained from an industrial forging process.

The structure of this paper is organised as follows: Section 2 details the industrial process used to test the efficiency of the developed thermal model, which is described in Section 3. The model includes the characterisation of thermal parameters and the specific formulations for the contact and spray-cooling phenomena. Section 4 details the numerical implementation strategy, while Section 5 presents the experimental methods for model validation. The main results are discussed in Section 6, including the validation analysis. Finally, Section 7 summarises the key findings, discussing their implications and outlining future lines of work.

## 2. Industrial Process Description

**Figure 1:**
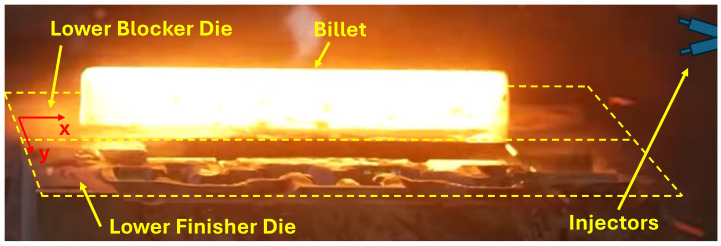
Side view of the lower blocker and lower finisher dies. Billet is placed on the lower blocker die. Location of the water sprays is sketched.

The industrial forging process to manufacture crankshafts at the CIE-Galfor company (https://cieautomotive.com/en/-/cie-galfor, accessed on 10 May 2025) involves two sequential press strokes: a preliminary stroke (using blocker dies) and a final stroke (finisher dies), as can be seen in Figure 1. The preliminary stroke, performed with the blocker dies, is the main deformation step, where the billet is shaped and most of the forming energy is applied. In contrast, the final stroke, carried out with the finisher dies, primarily serves to correct any minor surface imperfections and refine the final geometry. Since most heat generation and transfer occurs during the initial deformation, this study focuses on the thermal behaviour of blocker dies, which are subjected to higher thermal loads throughout the cycle.

The industrial forging process begins with the extraction of a 90 mm diameter cylindrical steel billet from an induction furnace. This billet is then transported along metallic guides by robotic arms and positioned on the lower blocker die (Stage #1). During this initial transport-and-placement phase, the billet begins to lose heat through natural convection and radiation. Simultaneously, the surrounding dies absorb a portion of this radiative energy.

Once the billet is properly located (Stage #2), it remains briefly on the lower blocker die before the forging impact. In this resting phase, heat transfer is primarily governed by conduction at the interface between the billet and the lower die. The forging impact follows (Stage #3), during which the upper die strikes the billet. This rapid deformation results in internal heating of the billet due to plastic work, while the dies experience additional heating through conduction and friction. Given the very short duration of this stage, the ambient thermal losses are negligible.

After deformation, the forged part is temporarily supported on the ejector pins (Stage #4). At this stage, the direct conduction path is interrupted, and the piece continues to lose heat through convection and radiation. The part is then transferred to the finisher die set (Stage #5), during which radiative heat from the crankshaft and convective losses are considered in the thermal model. Meanwhile, a cooling sequence for the dies begins.

Focusing on the dies cooling, first, a water–air spray is applied to the blocker dies, resulting in strong convective cooling (Stage #6). This is followed by lubrication (Stage #7), where a graphite–water–air mixture is projected to reduce the wear of the die in the next cycle. Finally, forced convection cooling continues as compressed air is blown over the dies (Stage #8), just before a new billet is inserted. Additionally, the incoming billet contributes to the thermal load on the dies via radiative heat transfer.

As can be seen, the process is divided into eight stages, each defined by dominant heat-transfer mechanisms. A concise description of each stage is presented below:

- **Stage #1**: The billet is transported to the lower blocker die. Heat loss occurs via natural convection and radiation, while the dies absorb radiative heat.
- **Stage #2**: The billet rests on the lower blocker die. Heat transfer is dominated by conduction at the contact interface, along with convection and radiation in non-contact regions. Figure 1 shows the billet placement.
- **Stage #3**: Forging impact. The dies are heated through contact and friction, while the billet experiences internal heating due to plastic deformation. Ambient losses are negligible, due to the very short duration.
- **Stage #4**: The forged piece rests on ejectors. Convection and radiation persist, but contact conduction disappears.
- **Stage #5**: Transfer of the piece to the finisher dies set. Average radiative heat from the crankshaft and convective losses are modelled.
- **Stage #6**: Water–air spray-cooling of blocker dies. Intense convective cooling occurs.
- **Stage #7**: Graphite lubrication. A graphite–water–air mixture is injected to reduce the blocker die wear.
- **Stage #8**: Air blow and new billet insertion. Forced convection cools the dies, while radiative heat from the incoming billet is considered.

It is worth noting that cycle durations are not strictly constant. Minor delays may arise due to malfunctions such as faulty electrovalves or insufficient pressure in actuator circuits. To account for this variability, more than ten consecutive cycles were monitored and analysed. The durations of each stage were then averaged, yielding a total cycle time of approximately 11.5 s.

## 3. Mathematical Model

The evolution of the thermal field in the forging dies and piece is governed by the classical heat conduction equation:

(1) $$\rho \left(T\right)c_{p}\left(T\right)\frac{\partial T}{\partial t}-\nabla \cdot \left(k\left(T\right)\nabla T\right)=Q\;\mathrm{in}\;\Omega$$

where *k*, ρ, and $c_{p}$ denote thermal conductivity, density, and the specific heat capacity of the solid material, while *T* stands for the temperature and *Q* represents the internal heat generation along the solid volume Ω. This heat source represents the heat generated by the deformation of the billet (it is null for the die’s domains).

Regarding material modelling, H13 tool steel (H13 (norm NADCA #207-2016), X40CrMoV5-1 (norm ISO 4957o [20])) was used as material for the dies, while 38MnVS5 steel (norm DIN EN 10267 [21]) was used for the piece. The materials’ properties dependency with temperature were defined as indicated in Table 1 and Table 2. Specifically, the properties for H13 steel were obtained from the company’s internal catalogue, whereas those for the billet material were sourced from the JMatPro database (https://www.sentesoftware.co.uk/jmatpro/, accessed on 10 May 2025).

For the billet/piece, the source heat *Q* generated by deformation during stage #3 is modelled by

(2) $$Q=\eta \left(\sigma :\dot{\varepsilon }\right)$$

where σ is the stress tensor and $\dot{\varepsilon }$ is the strain rate tensor. The parameter η represents the fraction of mechanical work converted into heat. For this problem, based on Rusinek et al. [22], we define η=0.9.

Therefore, during the piece deformation stage #3, the piece heat transfer problem is coupled to the mechanical problem, which, for the sake of brevity, is described in full detail in Appendix A.1.

### 3.1. Thermal Boundary Conditions

Equation (1) has to be solved using appropriate heat flux boundary conditions on the piece and dies surfaces Γ:

(3) $$-k\left(T\right)\frac{\partial T}{\partial \vec{\eta }}=q\;\mathrm{in}\;\Gamma$$

In Equation (3), $\vec{\eta }$ represents the unity normal vector to each (piece or die) surface location.

**Figure 2:**
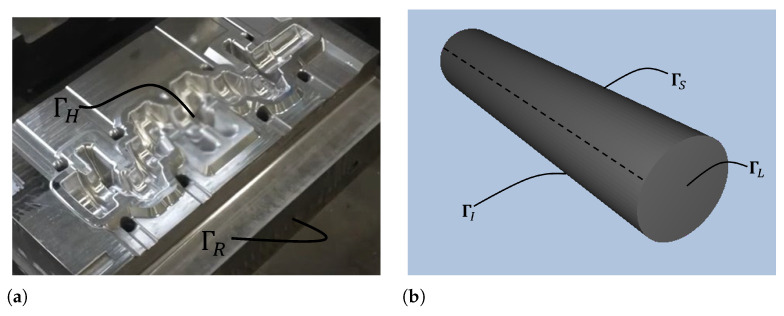
Boundary condition zones: (**a**) lower blocking die; (**b**) billet.

In this study, the billet/piece surface, as well as the dies impressions (see a schematic representation of the boundaries $\Gamma_{H}$, $\Gamma_{I}$, $\Gamma_{S}$, and $\Gamma_{L}$ in Figure 2), presented heat transfer through conduction, convection, and/or radiation, depending on the process stage. Thus, the heat flux *q* was divided as follows:

(4) $$q=q_{\mathrm{rad}}+q_{\mathrm{conv}}+q_{\mathrm{cont}}+q_{\mathrm{cool}}+q_{\mathrm{lub}}$$

where the following applied:

- $q_{\mathrm{rad}}$: radiative heat exchange;
- $q_{\mathrm{conv}}$: natural or forced convection cooling;
- $q_{\mathrm{cont}}$: heat transfer due to contact between the billet and the die;
- $q_{\mathrm{cool}}$: heat removed by spray-cooling;
- $q_{\mathrm{lub}}$: lubricant-induced heat transfer.

Table 3 summarises the heat transfer phenomena considered during each process stage.

The remaining die surfaces $\Gamma_{R}$ and the die surfaces in contact with the press were treated as adiabatic (q=0), due to minimal thermal exchange.

To capture the heat transfer interactions realistically, dedicated heat transfer models were developed and applied according to the dominant mechanism during each stage. These models enabled a stage-by-stage simulation of the thermal evolution along the dies and piece.

#### 3.1.1. Radiation: Heat Flux Model

In forging processes, where the billet spends time in close proximity to the dies—typically, a few centimetres away—or in direct contact, a significant portion of the radiation heat emitted by the billet is absorbed by the dies. Accurately accounting for this radiative heat transfer is essential to properly model the thermal evolution of the die surfaces.

However, the version of the forging simulation software employed in this study does not include a dedicated module to simulate radiative heat transfer between multiple solid surfaces. To overcome this limitation, a Surface-to-Surface (S2S) radiosity model was implemented using COMSOL (https://www.comsol.com/, accessed on 10 May 2025) Multiphysics. This method allows the calculation of net radiative fluxes by considering the mutual visibility, geometry, and emissivity of interacting surfaces.

The S2S model was applied to the die impression surfaces $\Gamma_{H}$ and the billet surfaces $\Gamma_{I}$ and $\Gamma_{S}$, which are the primary regions involved in radiative heat exchange during the forging process. The computed net radiative heat fluxes over these boundaries were later postprocessed and imposed as spatially varying thermal boundary conditions in the forging simulations. The complete numerical procedure used to compute and transfer these fluxes into FORGE is detailed in Section 4.1.

For the lateral billet surfaces $\Gamma_{L}$ radiation heat loss was modelled using the Stefan–Boltzmann law, assuming radiative exchange with the environment:

(5) $$q=-\varepsilon \sigma_{SB}\left(T_{\mathrm{billet}}^{4}-T_{\mathrm{air}}^{4}\right)$$

with $T_{\mathrm{air}}$ = 30 °C.

#### 3.1.2. Conduction: Contact Heat Transfer Model

The conductive heat transfer, active during stages #2 and #3, takes into account the contact heat transfer between the solids and the frictional heating, and it is modelled as follows:

(6) $$q_{\mathrm{cont}}=h_{\mathrm{cont}}\left(\sigma_{n}\right)\left(T_{\mathrm{die}}-T_{\mathrm{billet}}\right)+\frac{E_{\mathrm{billet}}\left(T\right)}{E_{\mathrm{billet}}\left(T\right)+E_{\mathrm{die}}\left(T\right)}\tau_{\mathrm{fr}}V_{\mathrm{slid}}$$

where $h_{\mathrm{cont}}$ is the contact heat transfer coefficient—in this case, dependent on the pressure $\sigma_{n}$, as explained below—$E\left(T\right)=\sqrt{k\rho c_{p}}$ is the effusivity, which is temperature dependent, while $\tau_{\mathrm{fr}}$ and $V_{\mathrm{slid}}$ are the shear stress and the sliding velocity between the solids at the evaluated surface location.

The contact heat transfer coefficient, $h_{\mathrm{cont}}$, is critical for conductive heat transfer and depends on factors such as temperature, pressure, roughness, lubrication, and material properties. Typically, $h_{\mathrm{cont}}$ is determined experimentally using methods like the Inverse Heat Conduction Method (IHTC), but due to the lack of direct experimental data in this study, we relied on the literature. Many studies have modelled $h_{\mathrm{cont}}$ for materials like aluminium [23], titanium alloys [24], and boron steels [25,26,27,28].

**Figure 3:**
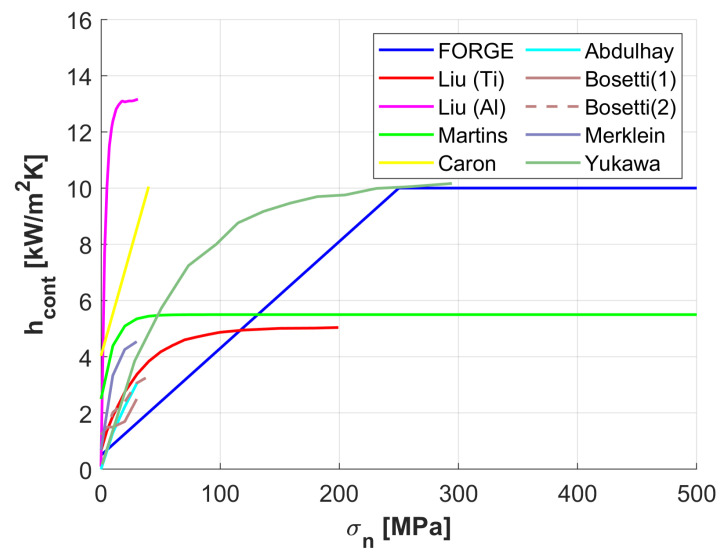
Comparative of $h_{\mathrm{cont}}$ values for different studies.

Among the influencing factors, contact pressure is the most significant; even a small increase in pressure can raise $h_{\mathrm{cont}}$ by up to $10\;\mathrm{kW}/m^{2}K$ [23]. As pressure increases, the microscopic contact area expands until plastic deformation nearly maximises the real contact area, causing $h_{\mathrm{cont}}$ to plateau. All the literature models of $h_{\mathrm{cont}}$ as a function of contact pressure ($\sigma_{n}$) are summarised in Figure 3, including the model used in FORGE NxT [29] and two other models described in the literature [30,31].

We developed a specific $h_{\mathrm{cont}}$ model based on the data from Liu et al. (2020) [24] for titanium $h_{cont,Ti}$. Using the Biot number, $Bi=\frac{hL}{k}$, and assuming equal characteristic lengths, we obtained, applying similarity, the heat transfer coefficient for the dies material H13,

(7) $$h_{cont}=h_{cont,Ti}\frac{k_{H13}}{k_{Ti}},$$

using the average conductivities $k_{H13}=24.4\;W/\mathrm{mK}$ and $k_{Ti}=20\;W/\mathrm{mK}$.

Additionally, the effect of lubrication between the two contact surfaces was also integrated into the model. In general, lubrication increases $h_{\mathrm{cont}}$ because lubricants typically have higher thermal conductivity than air, replacing air in the gaps and enhancing heat transfer. Moreover, lubricants help reduce oxidation, further improving $h_{\mathrm{cont}}$. However, as contact pressure increases, the gaps between the surfaces diminish, expelling excess lubricant and, thereby, reducing the incremental gain in $h_{\mathrm{cont}}$ [24].

**Figure 4:**
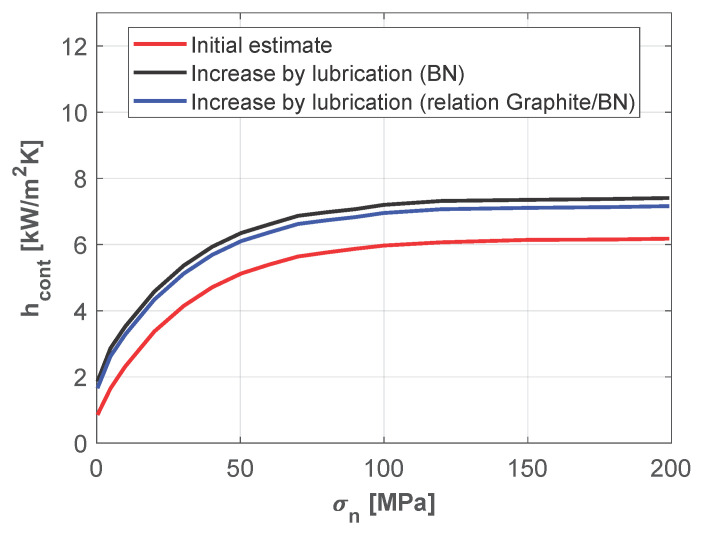
Dependency of $h_{\mathrm{cont}}$ with the contact pressure $\sigma_{n}$ (blue line).

Finally, the combined effect of lubrication on $h_{\mathrm{cont}}$ was incorporated into the model, with the results presented in Figure 4. The red curve represents the original $h_{cont}$ model based on the Biot number (without lubrication effects) given by (7). The black curve shows the model adjusted for lubrication with Boron Nitride (BN) according to the experimental data in [24]. The blue curve models the final enhancement due to a graphite solution by adjusting the lubrication curve with the ratio $k_{graphite}/k_{BN}$. This blue curve was the final model implemented in FORGE NxT.

#### 3.1.3. Convection: Water–Air Spray-Cooling Model

Modelling of the water–air spray-cooling stage #6 (and, to a lesser extent, the graphite–water lubrication stage #7) is critical, as a significant and rapid drop in the die surface temperature occurs.

There are two approaches to addressing these critical fast cooling stages: (i) performing a CFD simulation coupled with a solid heat transfer problem (also known as Conjugate Heat Transfer), or (ii) modelling and imposing a convective heat transfer coefficient $h_{\mathrm{cool}}$ in the cooling zone:

(8) $$q_{\mathrm{conv}}=h_{\mathrm{cool}}\left(T_{\mathrm{fluid}}-T\right)$$

where $T_{\mathrm{fluid}}$ is the surrounding fluid temperature and $T_{\mathrm{surface}}$ is the local surface temperature. Since FORGE NxT does not support the first option, and given its complexity, the second approach was chosen.

To characterise the spray-cooling HTC, the most common method is to heat a material sample (with a simple geometry) to a specific temperature and monitor its thermal evolution during cooling, using thermocouples, and to solve, afterwards, an Inverse Heat Conduction Problem (IHCP). However, small experimental disturbances can cause problems during characterisation, which is why some researchers focus on improving this type of analysis [32]. Also, the heat transfer on the surface die differs from the HTC values obtained from this type of simple experiment, as the die impression is geometrically complex, with cavity surfaces having recesses and regions that are beyond the reach of the spray impact.

This HTC depends on multiple factors:

- Impact density $\rho_{imp}$. This parameter is the most influential [33] and represents the mass flow rate (${\dot{M}}_{w}$) impacting a given area ($A_{imp}$):
  (9) $$\rho_{imp}=\frac{{\dot{M}}_{w}}{A_{imp}}\left[\frac{\mathrm{kg}}{m^{2}s}\right]$$
  The spatial distribution of $\rho_{imp}$ depends on the type and geometry of the injector. In jet cooling systems, the flow is concentrated in narrow areas, maximising local $h_{\mathrm{cool}}$ but with limited coverage. Conversely, spray-cooling (used in this study) distributes the fluid more homogeneously, sacrificing cooling intensity for uniformity [34]. The typical distribution follows a bell-shaped profile, with a maximum $\rho_{imp}$ at the jet axis and radial decay (see [33] for an example of the flow distribution).
- Surface temperature *T*. According to Czechowski et al. (2003) [35], four thermal regimes are identified: (1) Liquid Convective, where $T<T_{evaporation}$ and cooling occurs via natural convection; (2) Nucleate Boiling, where $T>T_{evaporation}$ and bubble formation enhances heat transfer; (3) Transition Boiling, where a partial vapour layer gradually reduces thermal transfer; and (4) Film Boiling, in which a continuous vapour film insulates the surface, minimising $h_{\mathrm{cool}}$.
- Droplet size. According to Anisiuba et al. (2021) [36], high atomising air pressures reduce droplet size and increase droplet velocity, facilitating penetration into the vapour layer forming on the surface, although this effect diminishes at high water flow rates. Similarly, Liu et al. (2018) [37] demonstrated that small droplets—generated by high pressure—evaporate more rapidly, thereby increasing the heat transfer coefficient. These mechanisms support models such as that of Kortabeck et al. (2021) [38], which incorporate pressure as a predictive variable due to its influence on droplet size.

Additional models that include the influence of all these parameters are the following: models based on temperature, like Hadala et al. (2019) [39], models based on droplet size [40], models based on impact density [41,42,43], and also models that account for pressure effects [44], among others.

In our case, we opted to implement a modified version of the Bocanegra et al. (2021) model [45], as it integrates all these effects into a single expression. This model [45] is defined as

(10) $$h_{\mathrm{cool}}=379.93\;\rho_{\mathrm{imp}}^{0.318}\;u_{z}^{0.33}\;T_{\mathrm{surface}}^{-0.895}\;d_{30}^{-0.024}$$

where $h_{cool}$ is in kW/(m^2^ K), $\rho_{imp}$ is in L/m^2^ s, and $u_{z}$ and $d_{30}$ represent the droplet velocity normal to the surface and the mean droplet diameter, set to $u_{z}=11$ m/s and $d_{30}=65$ µm, respectively [45]. The local surface temperature *T* is obtained from the simulation itself, while the impact density $\rho_{imp}$, instead of assuming a constant value, is modelled using a spatial distribution based on the specific characteristics of the problem.

##### Impact Density Model

**Figure 5:**
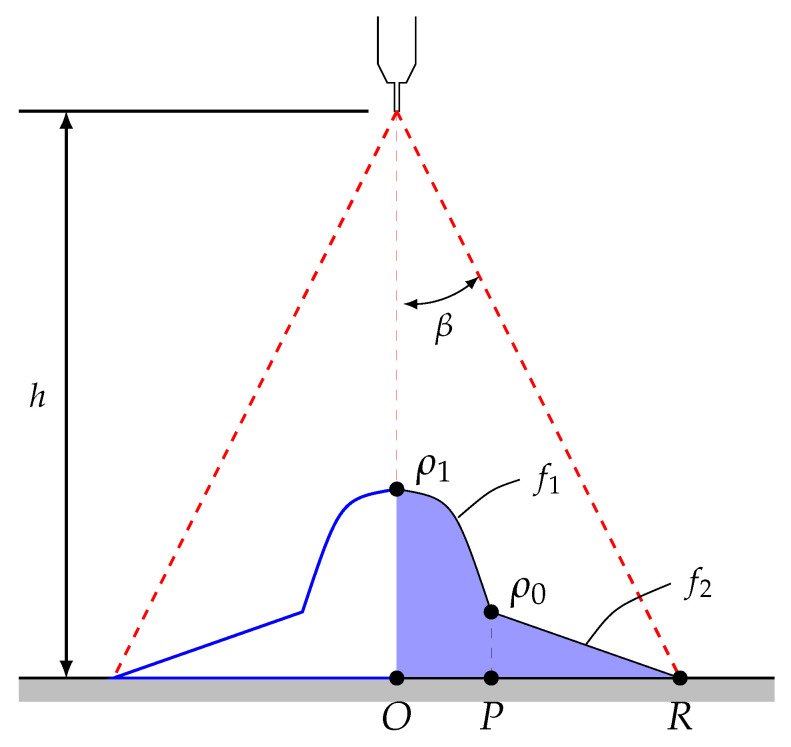
Impact density distribution model.

The impact density distribution $\rho_{\mathrm{imp}}\left(r\right)$ is defined using two functions, $f_{1}$ (parabolic) and $f_{2}$ (linear), which depend on the radial position *r*, as indicated in Figure 5. These distribution functions are detailed in Equation (11):

(11) $$\left.\begin{array}{c} f_{1}\left(r\right)=\rho_{1}-k\cdot r^{2},\;r\leq P\;\\ f_{2}\left(r\right)=\rho_{0}\left(1-\frac{r-P}{R-P}\right),\;r>P. \end{array}\right\}$$

where $k=\frac{\rho_{1}-\rho_{0}}{P^{2}}$ and R=htan(β). The variable *R* represents the end point of the wetted area, with β being the maximum spray angle of the injector (typically provided by manufacturers) and equal to $16.5^{\circ }$ in the industrial process. Distance *h* is set to 200 mm. Parameters $\rho_{0}=\alpha \rho_{1}$ and P=ωR are, in turn, defined by setting the percentage parameters α=0.1 and ω=0.2, characterised by Liu et al. (2020) [33].

Parameter $\rho_{1}$ is then determined, imposing mass conservation; that is, the known injector’s mass flow rate (${\dot{M}}_{w}=0.15\;\mathrm{kg}/s$) equals the mass flow received by the wetted area (${\dot{M}}_{w}={\dot{M}}_{1}+{\dot{M}}_{2}$, where ${\dot{M}}_{1}$ and ${\dot{M}}_{2}$ correspond to the mass flow rates under $f_{1}$ and $f_{2}$, respectively). The flows are calculated as follows:

(12) $${\dot{M}}_{1}=\int_{0}^{2\pi }\int_{0}^{P}\left(\rho_{1}-\frac{\rho_{1}-\rho_{0}}{P}r^{2}\right)r\;dr\;d\theta =\frac{\pi P^{2}}{2}\left(\rho_{1}+\rho_{0}\right)$$

(13) $${\dot{M}}_{2}=\int_{0}^{2\pi }\int_{P}^{R}\rho_{0}\left(1-\frac{r-P}{R-P}\right)r\;dr\;d\theta =2\pi \rho_{0}\cdot \frac{1}{R-P}\left(\frac{R}{2}\left(R^{2}-P^{2}\right)-\frac{1}{3}\left(R^{3}-P^{3}\right)\right)$$

Finally, the following expression is obtained:

(14) $${\dot{M}}_{w}={\dot{M}}_{1}+{\dot{M}}_{2}=\frac{\pi R^{2}\rho_{1}}{6}\left(\omega (3\omega -\omega \alpha +2\alpha )+2\alpha \right)$$

Thus, solving the above expression provides Equation (15) to compute $\rho_{1}$. Note that FORGE NxT uses units in millimetres, so we must multiply by $10^{6}$ to convert $\rho_{1}$ into [kg/m^2^s]:

(15) $$\rho_{1}=\frac{6\cdot 10^{6}{\dot{M}}_{w}}{\pi R^{2}\left(\omega (3\omega -\omega \alpha +2\alpha )+2\alpha \right)}\left[\frac{\mathrm{kg}}{m^{2}s}\right]$$

This model was incorporated as a new cooling module inside FORGE NxT 3.2.

Graphite lubrication stage #7, in which a graphite-based fluid is sprayed over the die surfaces, induces a certain level of heat extraction. However, as the primary purpose of this stage is to reduce friction rather than to cool the dies, the associated thermal effect is relatively mild when compared with the water spray stage. Although the literature on lubricant-induced thermal behaviour is limited [46,47], a conservative constant heat transfer coefficient of $h_{\mathrm{cool}}=500$ W/m^2^K was adopted. Sensitivity analyses confirmed its limited influence on die temperature, in line with its secondary role in thermal regulation.

Finally, for the process stages #1–#5 and #8, convective cooling was modelled, assuming natural convection with ambient air at 30 °C by using the Heat Transfer Coefficient (HTC) $h_{\mathrm{cool}}=10$ W/m^2^K reported in the literature for similar geometries [48].

### 3.2. Initial Conditions

**Figure 6:**
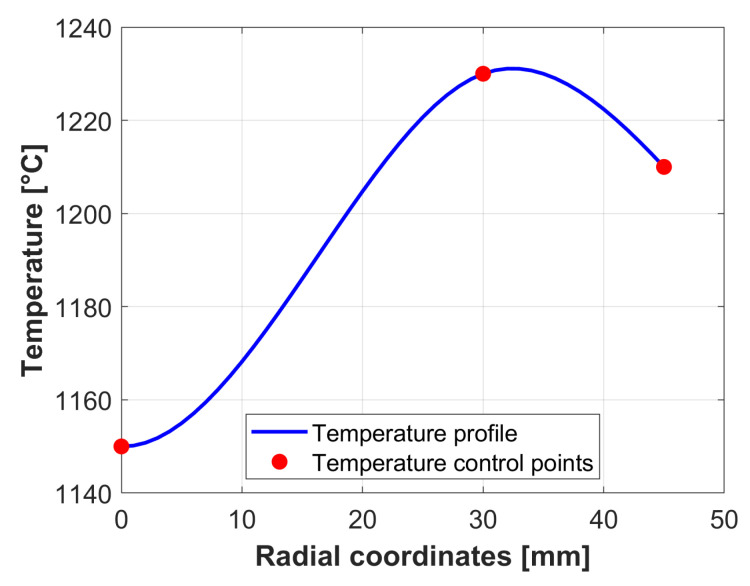
Temperature distribution of the billet: Billet centre (radial coordinate 0), billet surface (radial coord. r=45 mm).

For the upper and lower dies a constant initial temperature of 130 °C was set, as they are preheated to this temperature before beginning the process. The initial billet’s radial temperature distribution was obtained from a previous simulation of the billets heating inside the induction furnace. Thus, the initial radial temperature distribution shown in Figure 6 was applied to the billet.

## 4. Numerical Implementation

To solve the problem, the commercial software FORGE NxT 3.2 was used. This tool is specifically designed for the analysis of thermomechanical processes involving material deformation. It allows the simulation of each stage of the process, including both thermal and mechanical phenomena.

In this work, the steady-state thermal module was selected, as the objective was to determine the repetitive thermal field reached by the dies set during production. This module first solved the transient thermal problem defined by Equation (3) for each stage, and, once all stages had been computed, it derived the repetitive temperature distribution during production based on those results. As a result, it allowed for the evaluation of both the temperature field at the end of each individual stage and the overall transient thermal evolution throughout the process.

For stage #3 (forging impact on the billet), the mechanical model (described in Appendix A.1) was solved coupled to the thermal model described in Section 3. The numerical implementation of this thermal–mechanical coupling is described in detail in Appendix A.2.

### 4.1. Data Preprocessing: Radiation Heat Fluxes

As mentioned in Section 3.1.1, radiative heat transfer plays a dominant role during various process stages when the billet is near the dies. However, since FORGE NxT 3.2 does not support multi-body radiation modelling, the net radiative heat fluxes had to be computed externally and applied as spatially varying boundary conditions in the simulation.

To achieve this, an auxiliary simulation was carried out in COMSOL Multiphysics, using a simplified geometry that captured the key features of the billet–die configuration. The interacting surfaces—billet and die impressions—were discretised into approximately 20 surface elements each, allowing for accurate computation of view factors.

A Surface-to-Surface (S2S) radiosity model was used to evaluate the net radiative exchange between the billet and the die surfaces. Emissivity values of 0.88 for the billet and 0.6 for the dies were applied. Solving the radiosity system yielded the spatial distribution of radiative heat fluxes for each relevant process stage.

**Figure 7:**
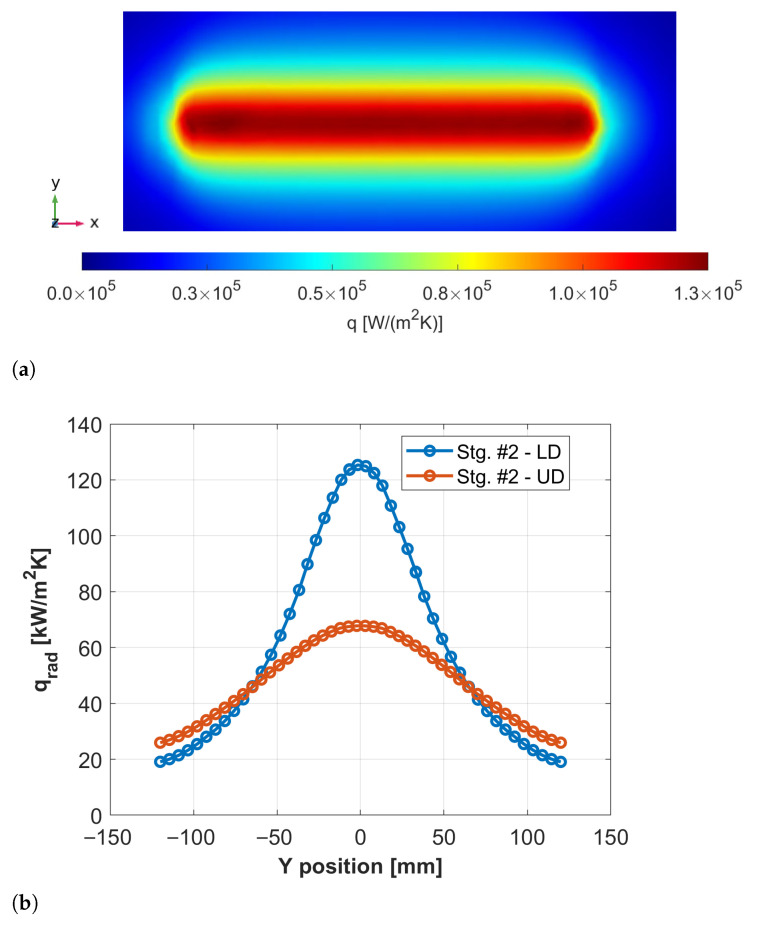
(**a**) Net radiation flux received by the lower blocker die during stage #2. (**b**) Plot of the heat flux distribution along the Y-axis for the lower blocker die (blue) and upper blocker die (red).

**Figure 8:**
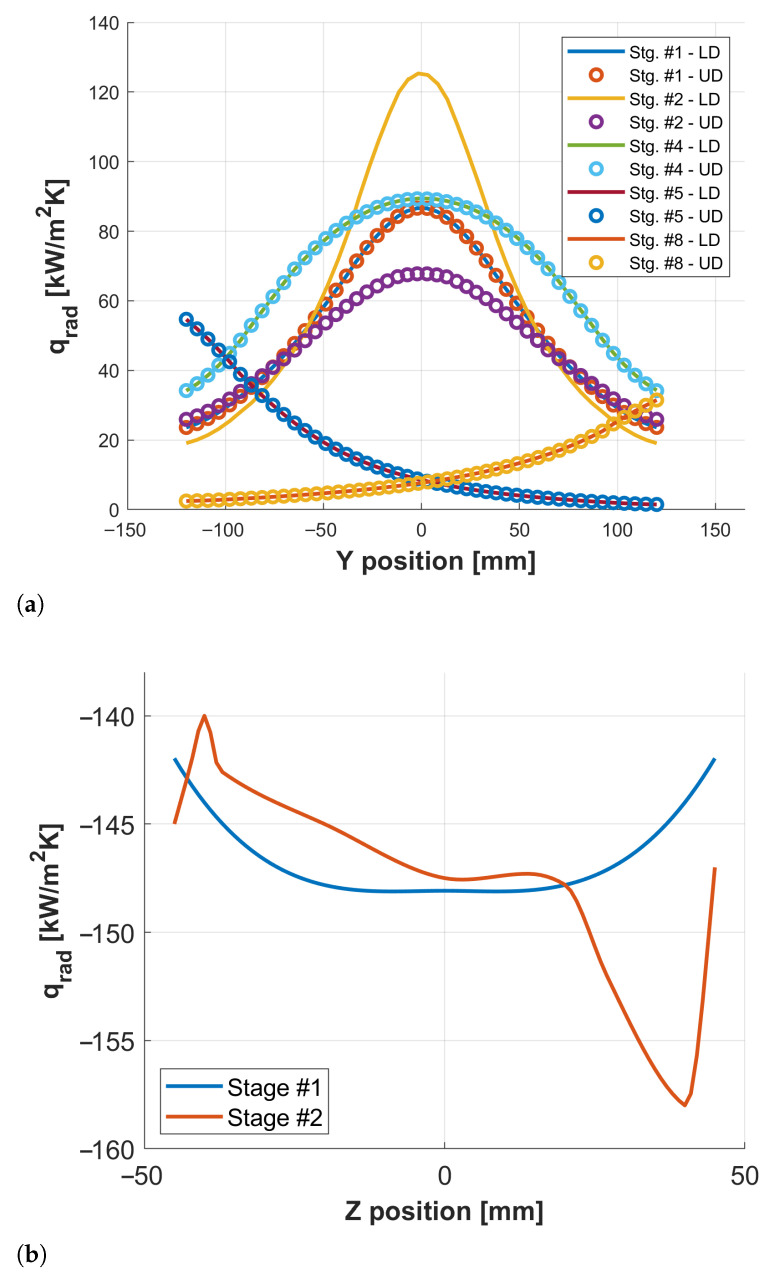
(**a**) Radiative fluxes distribution with coordinate *Y* along the symmetry plane (for the upper and lower blocking dies) during stages #1, #2, #4, #5, and #8. (**b**) Billet radiative fluxes distribution with the vertical coordinate *Z* (being Z=0 the center of the billet) during stage #1 and #2.

Examples of these distributions are presented in Figure 7 and Figure 8. Figure 7a shows the net radiative flux received by the lower blocker die during stage #2, which exhibits a peak at the centre and a gradual decrease along the Y-axis, due to the billet’s geometry and distance. Figure 7b compares the flux distributions between the upper and lower dies in the same direction.

Figure 8a illustrates the flux profiles along the symmetry axis (Y-direction) for stages #1, #2, #4, #5, and #8, revealing significant variations depending on billet positioning. Additionally, Figure 8b shows the vertical (Z-axis) flux distribution emitted by the billet during stages #1 and #2, with Z=0 corresponding to the billet mid-height. This information is essential for stage #3, where the thermal field of the billet must be known to accurately compute heat transfer by conduction to the dies.

The extracted profiles were processed using a custom Python script and formatted for integration into FORGE NxT, where they were applied as non-uniform boundary heat fluxes on the corresponding surfaces.

### 4.2. Geometry and Mesh

The CAD models of the dies, provided by CIE-Galfor company, were imported into the software. For the billet, a solid cylinder 90 mm in diameter was created. The meshing of all the geometries was performed using the software’s internal meshing tool. For the thermal problem the software employs a P1 element, whereas for the mechanical problem a P1/P1 formulation is used. The number of elements and nodes for each object is detailed in Table 4.

A mesh sensitivity analysis was also carried out. First, the stage most sensitive to mesh refinement was identified, which turned out to be the billet deformation stage. This stage involves complex contact interactions, and mesh size has a strong influence on the accuracy of the solution. For this reason, the analysis focused on the average surface temperature of the first two contact points between the billet and the blocker die, evaluated immediately after contact.

**Figure 9:**
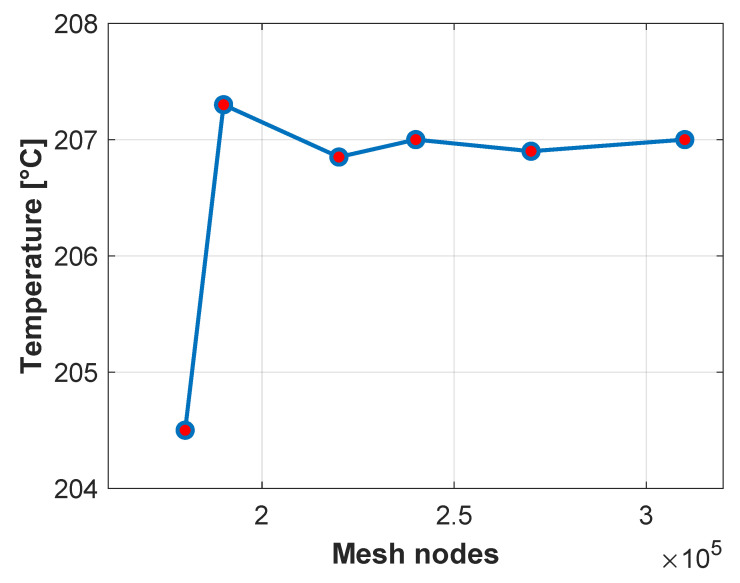
Mesh sensitivity analysis. Averaged surface temperature of the first contact points between the billet and the blocker die versus number of die mesh nodes.

Simulations were performed using meshes ranging from 175,000 to 300,000 nodes in the dies. It was observed that coarse meshes resulted in significantly underestimated temperatures. As the mesh was refined, the temperature predictions increased until they stabilised at approximately 207 °C. It was found that beyond 240,000 nodes the temperature values in the evaluated contact region showed negligible variation, indicating solution convergence. Figure 9 shows the averaged thermal evolution obtained for these different meshes.

### 4.3. Time Step Adjustment

For the present simulations, a maximum time step of Δt=0.1 s and a maximum thermal increment of ΔT=2.5 °C were set across all stages. Thus, the software automatically adjusted the time step to ensure that the temperature change at any node did not exceed the defined limit. This configuration provided stable and accurate results without requiring further refinement. However, during thermomechanical stage #3, the time step is automatically managed by the software, due to the complexity of the mechanical problem, which demands significantly smaller time increments. The thermal problem during this stage adopted the time step set by the mechanical model, which was equal, on average, to $\Delta t=8.92\times 10^{-4}$ s. Regarding numerical convergence, a time iteration is converged when the ratio between the current residual and the initial residual is lower than $10^{-7}$.

To optimise computational performance, FORGE NxT utilises parallel computing and preconditioning techniques. In this work, simulations were run using 6 cores. The computations were performed on a high-performance workstation equipped with 128GB of RAM, dual Intel Xeon Silver processors (12 cores total). The computational time to reach the repetitive state was 12 h.

## 5. Experimental Methods

For validation purposes, a thermal camera PCE-TC 31 (PCE Instruments, Meschede, Germany https://www.pce-instruments.com/, accessed 10 May 2025) was employed to capture the surface temperature distribution of the lower blocker die. The PCE-TC 31 camera has a thermal sensitivity of 0.1 °C (at 30 °C) and a measurement accuracy of ±2 °C or ±2% within its operating range of −20 °C to +350 °C. This level of precision is suitable for validating the temperature fields in forging dies. Prior to measurements, the emissivity of the camera was calibrated by using the billet as a reference object. Specifically, the temperature of the billet was measured at the exit of the induction furnace, where the surface temperature was known and its emissivity was approximately 0.88. The thermal camera settings were adjusted until its readings matched this reference, ensuring consistency in subsequent measurements.

**Figure 10:**
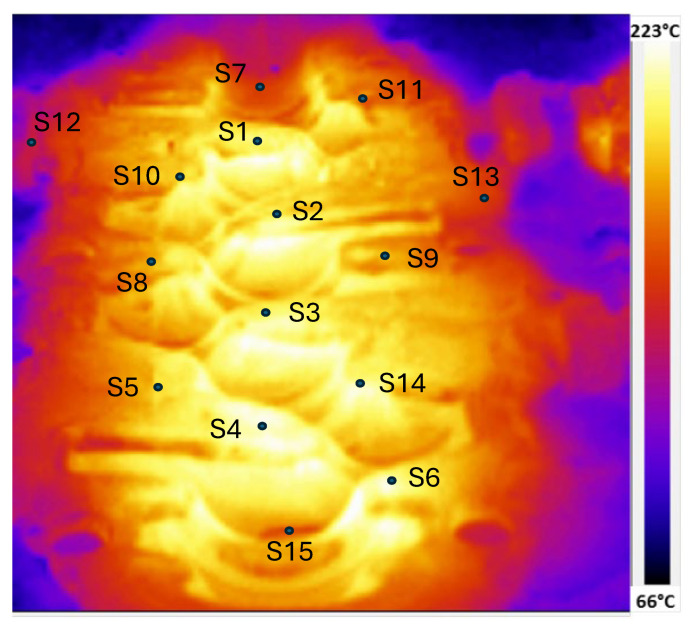
Thermal camera measurements of the steady-state thermal field for the lower blocker die. Location of analysed measurement points are indicated in the figure.

After two hours of continuous operation—allowing the thermal field to reach steady-state conditions—thermographic images of the die were taken. The analysis focused on 15 specific surface points (S1 to S15), which are clearly marked in the thermal image (see Figure 10).

## 6. Results and Discussion

### 6.1. Model Validation

**Figure 11:**
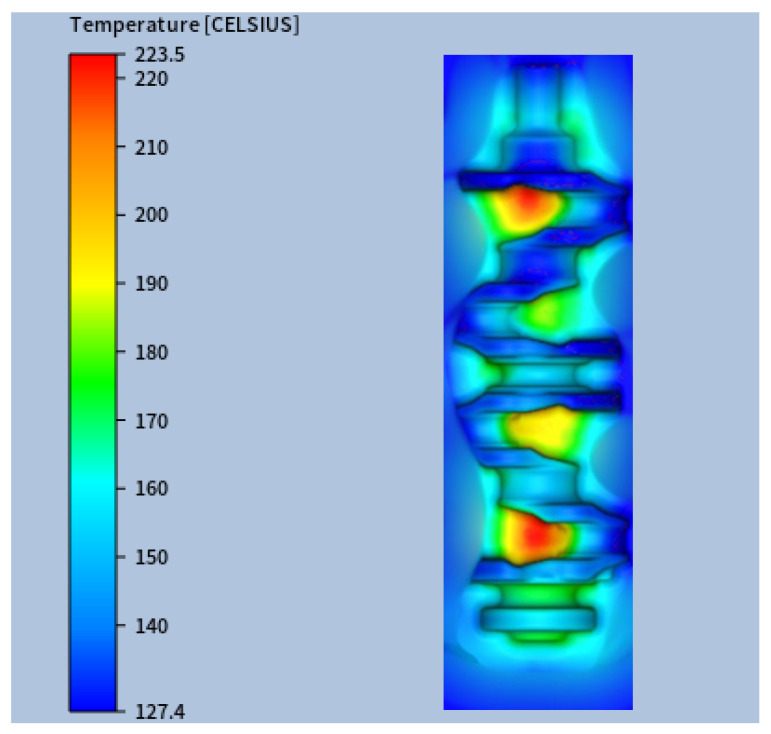
Numerical steady-state thermal field for the lower blocker die.

The experimental values were then compared to the numerical predictions (see Figure 11, which, for confidentiality reasons, shows only part of the die impression surface). This comparison allowed for a detailed evaluation of the model’s ability to reproduce the actual thermal conditions experienced by the forging dies during operation. The numerical results were extracted once the system reached its steady-state thermal regime, ensuring consistency with the experimental measurements, which were recorded under steady-state production conditions.

To assess the agreement quantitatively, temperature values at selected locations were compiled and are presented in Table 5. The comparative results show that the numerical predictions closely match the experimental data across all measured points. The average relative error was 5.80%, with a maximum deviation of 13.7% observed at location S2.

### 6.2. Analysis of the Steady-State Thermal Field

**Figure 12:**
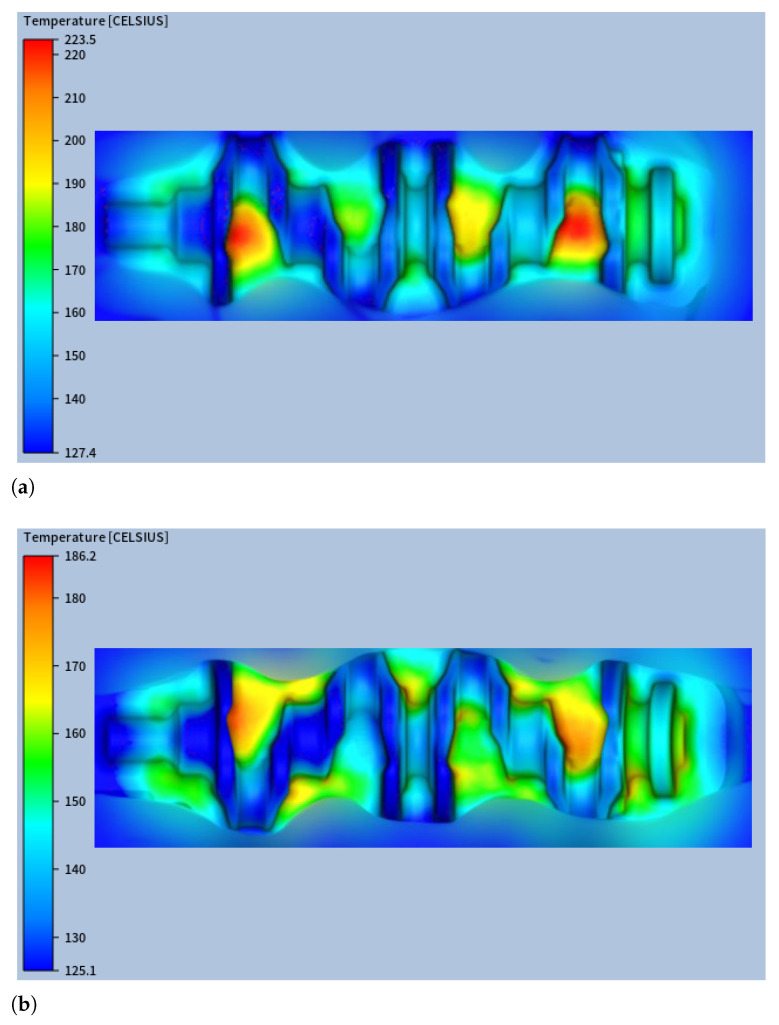
Steady-state thermal field for (**a**) lower blocker die and (**b**) upper blocker die.

Having confirmed the accuracy of the numerical model through experimental validation, the simulation results were further analysed to gain deeper insights into the thermal behaviour of the forging dies under steady-state operating conditions. The thermal analysis revealed several key phenomena related to the temperature distribution within the forging dies. As expected, the lower blocker die consistently reached higher temperatures than the upper die, as shown in Figure 12 (for confidentiality reasons, Figure 12 shows only part of the die impression surface), with differences (on the impression surface) in the maximum temperature and in the average temperature of 37.3 °C and 15.1 °C, respectively.

This behaviour can be primarily attributed to two main factors. First, during stage #2 of the process—when the billet is in contact with the lower blocker die—four distinct contact regions form, each reaching an average temperature of approximately 225 °C. These localised hotspots contribute significantly to the overall heating of the surrounding areas. Secondly, radiative heat transfer also plays a relevant role. As the billet approaches the lower die during stage #2, the radiative exchange is more intense toward the lower die than the upper one. This imbalance further contributes to the observed asymmetry in the thermal fields.

Additional insights from the simulations indicate that some regions of the die tended to overheat due to limited exposure to the cooling system. Specifically, the geometrical configuration of the die surfaces created areas that were partially shielded from direct spray-cooling. These zones exhibited higher temperatures, suggesting the need for improved cooling strategies to enhance temperature uniformity and reduce overall cooling times.

**Figure 13:**
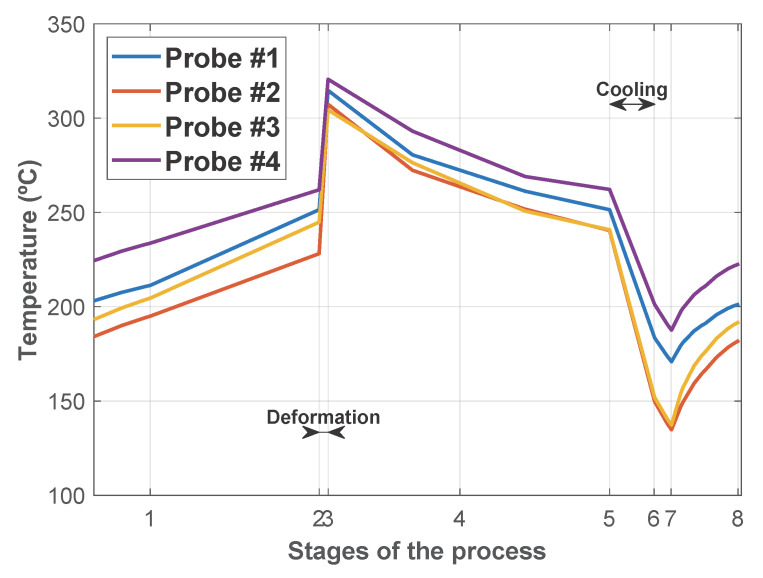
Temperature evolution at different locations in the lower blocker die during the stages of the process.

Once the repetitive thermal state was reached, the temperature at points S1, S2, S3, and S4 (see locations in Figure 10) along the whole simulated cycle was plotted in Figure 13. A noticeable temperature rise occurred during the deformation stage (Stage #3). In contrast, the cooling stages effectively reduced the temperature, helping to manage and mitigate thermal accumulation in the dies.

**Figure 14:**
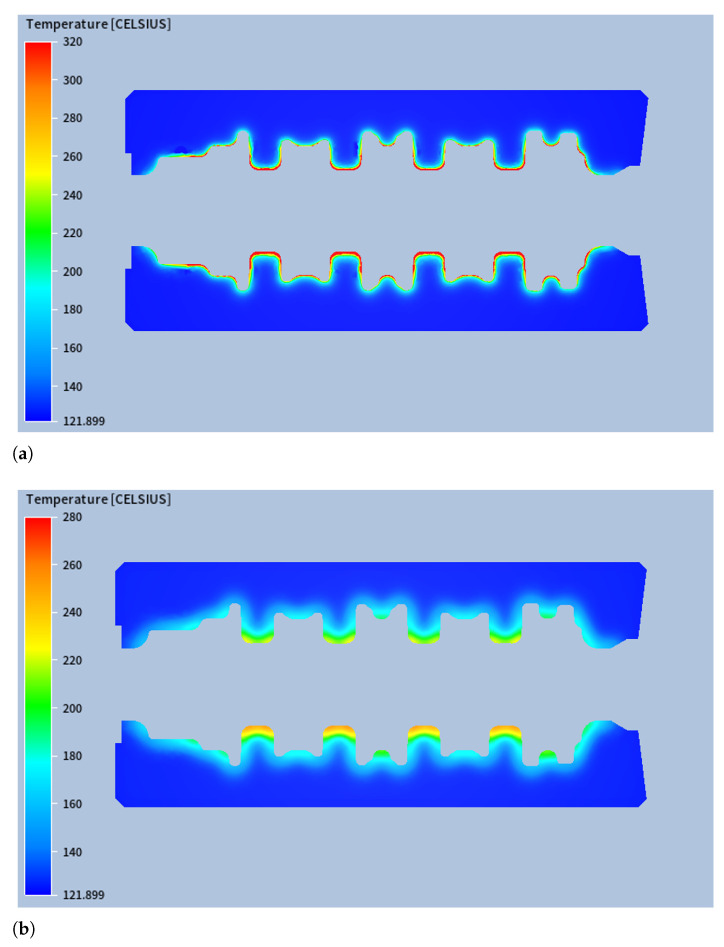
Temperature field on a mid-plane (**a**) at the beginning of stage #4 and (**b**) at the end of stage #5.

It is notable that the surface temperature of the die matrices at the end of Stage #5 was lower than at the beginning of Stage #3, even though radiative heat flux was still present during this period. However, a cross-sectional analysis revealed that, in this case, the conductive heat flux into the interior of the matrices exceeded the incoming radiative heat flux at the surface. During Stages #4 and #5, the high temperature gradients established in Stage #3 drove a significant conductive heat flux from the surface toward the interior. This resulted in surface cooling as the internal regions gradually heated up. Figure 14a,b show cross-sectional temperature fields at the start of Stage #4 and the end of Stage #5, respectively, illustrating the diffusion of heat from the surface into the bulk material.

### 6.3. Influence of Stage Duration on the Die Thermal State

Finally, targeted simulations were conducted to assess the effect of modifying the duration of the critical stages—specifically, stages #2 (resting of the billet on the lower blocker die) and #6 (water spray-cooling). These tests, shown below, confirmed that altering the duration of the billet contact or the spray-cooling (by increasing the first and reducing the second by 20%, approximately) affects the resulting thermal field. Additionally, an analysis of the radiative effects showed that radiation during the pre-impact stage plays a non-negligible role in the overall heating of the lower die.

#### 6.3.1. Influence of Billet Resting Time on Lower Blocker Die (Stage #2)

**Figure 15:**
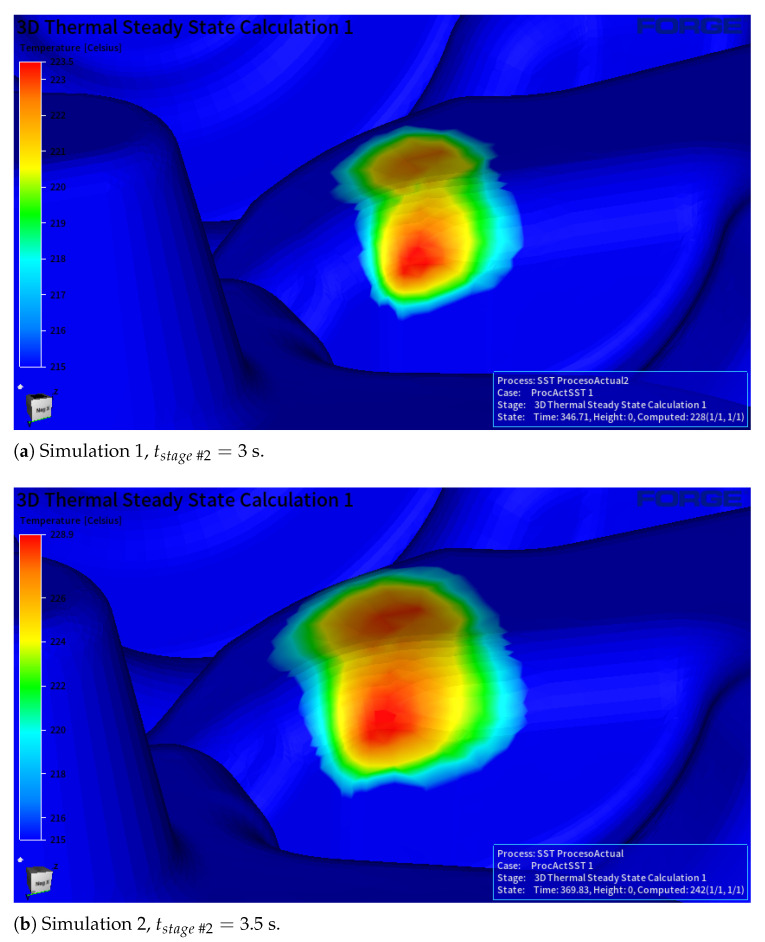
Hot zone region in the steady-state thermal field.

Stage #3 (impact phase) is the most critical, as high contact pressures between the billet and lower die generate elevated heat transfer coefficients ($h_{cont}$) and surface temperatures. Since contact time in stage #2 (billet support) is the only adjustable parameter, two full-cycle simulations of different durations were conducted for this stage #2 time: 3 s (Simulation 1) and 3.5 s (Simulation 2). Figure 15 compares the sizes of a hot zone at the end of stage #2, while Table 6 shows that increasing the resting time by 17% increases both mean and maximum temperatures by approximately 6 °C. Additionally, the table shows the time needed to reach the repetitive thermal state along the production. The simulation with more contact time needed 24 s more (i.e., production of three pieces more) to reach this thermal-field stability.

#### 6.3.2. Influence of Water Spray-Cooling Time (Stage #6)

**Figure 16:**
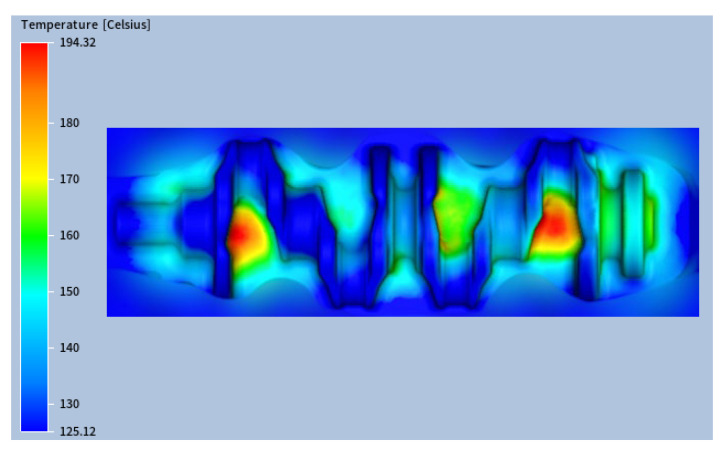
Steady-state thermal field with $\left(t_{stage\;\#6}=1.4\right)$ s.

The cooling stage plays a critical role in reducing the die temperature. In addition to the baseline case shown in Figure 12a (with a cooling time of 1.1 s), an additional simulation was performed with an increased cooling time of 1.4 s to evaluate its effect on the thermal cycle. As shown in Figure 16 (for confidentiality reasons, Figure 16 shows only part of the die impression surface), increasing the cooling time by 21% led to a reduction of the maximum die temperature to 194.3 °C, which represented a 13% decrease compared to the original case.

## 7. Conclusions

This work has presented a comprehensive thermal modelling approach for forging dies, addressing the complexity of heat transfer throughout the various stages of the process. Each stage was carefully analysed and assigned specific boundary conditions, allowing for a detailed and realistic simulation of the thermal behaviour of the dies during the forging cycle. The modelling approach required the development and integration into the commercial software FORGE NxT of two distinct heat transfer models: a thermal contact model between the billet and the dies, and a water spray-cooling model used in the process.

The thermal contact model was formulated based on contact pressure and included the effect of lubrication on the heat transfer coefficient, which plays a critical role in accurately capturing the heat exchange at the interface. An air–water spray distribution model was also developed and integrated to simulate the spray-cooling system applied between cycles. These models were grounded in the existing literature data, and a significant research effort was dedicated to reviewing and selecting the most appropriate formulations for each stage of the forging process. The simulation results were validated against experimental thermal images extracted from a crankshaft hot forging process, yielding an average relative error of 5.8% in the thermal state during production.

Compared to the simplified models typically used in industrial software such as FORGE—which assume a uniform water distribution over the sprayed area—the enhanced formulations implemented here significantly improve prediction accuracy by capturing the non-uniformity of water concentration, particularly its higher intensity near the jet axis. This leads to a more realistic representation of the cooling process. The model is robust, easy to implement, and can be used to analyse the impact of various production parameters on the industrial process, providing valuable insights for hot forging operations.

Ongoing efforts are focused on enhancing the thermal models through experimental validation. More detailed physical testing will be conducted to refine both the contact and cooling models, ensuring that they better reflect real-world conditions. Furthermore, considering that the forging process is highly standardised, and that, generally, only the spray-cooling time can be adjusted, a predictive model based on Proper Orthogonal Decomposition (POD) is being developed (see different examples of use in [49,50,51]). This enhanced model aims to forecast in short times (of less than 1 s) the thermal field as a function of the cooling duration, allowing for better process control and optimisation.

In parallel, future studies will address the thermal behaviour in alternative forging setups involving different die geometries and billet sizes—for instance, configurations designed to reduce flash formation, which typically employ smaller billets. Applying the proposed models to these new scenarios will also allow for assessing their robustness and effectiveness when dealing with different geometrical configurations. These developments are expected to further improve product quality and tool life, while contributing to the broader goal of implementing data-driven control strategies in traditional manufacturing processes.

## Figures and Tables

**Table 1:** Density and thermal properties for the billet material (Steel 38MnVS5).

*T* [°C]	ρ [kg/m^3^]	*k* [W/mK]	$c_{p}$ [J/kgK]
800	7605.2	26.7	605.1
850	7577.5	27.4	615.2
900	7549.9	28.0	623.8
950	7522.4	28.6	631.0
1000	7495.1	29.3	639.3
1050	7467.8	29.9	649.3
1100	7440.6	30.5	660.7
1150	7413.6	31.2	653.6
1200	7386.8	31.8	661.9
1250	7360.1	32.4	670.2
1300	7333.5	33.1	678.9
1350	7306.8	33.7	687.7
1400	7280.1	34.3	696.9

**Table 2:** Counterpart of Table 1 for the dies material (Steel H13).

*T* [°C]	ρ [kg/m^3^]	*k* [W/mK]	$c_{p}$ [J/kgK]	*E* [GPa]
20	7800	25	460	210
400	7700	29	550	180
600	7600	30	590	140

**Table 3:** Heat transfer phenomena active at the blocking die surfaces and the billet/piece surfaces during each process stage. The symbol L indicates lower die, U indicates upper die, and B indicates billet/piece. A dash (–) indicates the phenomenon is not present in that stage.

Stage	$q_{\mathrm{rad}}$	$q_{\mathrm{conv}}$	$q_{\mathrm{cont}}$	$q_{\mathrm{cool}}$	$q_{\mathrm{lub}}$
#1	L,U,B	L,U,B	–	–	–
#2	L,U,B	L,U,B	L,B	–	–
#3	–	–	L,U,B	–	–
#4	L,U	L,U,B	–	–	–
#5	L,U	L,U,B	–	–	–
#6	–	–	–	L,U	–
#7	–	–	–	–	L,U
#8	L,U	L,U,B	–	–	–

**Table 4:** Characteristics of the employed meshes.

Geometry	Size Element [mm]	Number of Elements (Tets.)	Nodes
Billet	4.5	75,000	16,975
Lower Die	15	1,195,152	240,682
Upper Die	15	1,189,043	239,913

**Table 5:** Comparison between real and numerical temperatures with relative error per location.

Probe	Real Temperature (°C)	Numerical Temperature (°C)	Relative Error (%)
S1	206.3	201.4	2.38%
S2	211.0	182.1	13.70%
S3	206.0	192.0	6.80%
S4	219.4	221.5	0.95%
S5	196.7	173.0	12.05%
S6	205.6	196.8	4.28%
S7	156.2	160.8	2.86%
S8	182.3	166.7	8.56%
S9	180.4	161.1	10.70%
S10	187.1	191.4	2.25%
S11	169.1	159.6	5.62%
S12	136.4	132.7	2.71%
S13	156.6	160.8	2.61%
S14	187.7	203.1	7.58%
S15	160.5	154.2	3.93%
Average Relative Error			5.80%

**Table 6:** Analysis of the effect of time duration of stage #2 on the temperature of the hot points.

	Simulation 1 ($t_{\mathrm{stage}\;\#2}=3$ s)	Simulation 2 ($t_{\mathrm{stage}\;\#2}=3.5$ s)
$T_{mean}$ [°C]	220.5	226
$T_{max}$ [°C]	223	228.9
$t_{repetitive\;state}$ [s]	346.7	369.8

**Figure A1:**
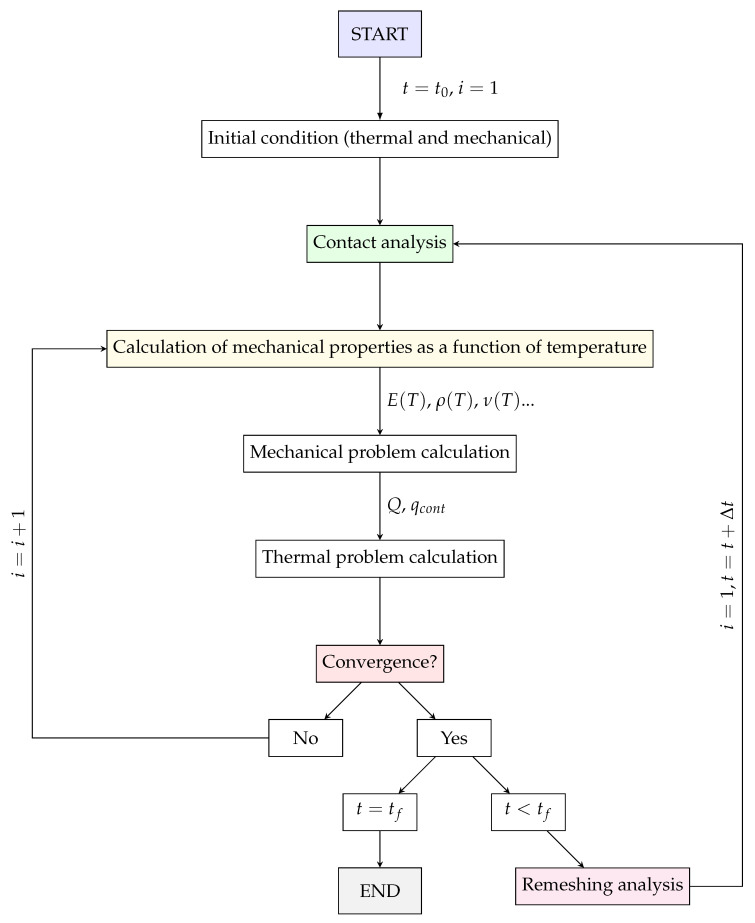
Coupling thermal–mechanical algorithm implemented in FORGE NxT software.

## Data Availability

The original contributions presented in the study are included in the article, further inquiries can be directed to the corresponding author.

## Abbreviations

The following abbreviations are used in this manuscript:

FEM	Finite Element Method
POD	Proper Orthogonal Decomposition
ML	Machine Learning
AI	Artificial Intelligence

## Appendix A. Mechanical Problem and Thermomechanical Coupling

During the first forging stroke, stage #3, the mechanical model is needed to compute the heat *Q* generated during deformation and friction, the contact pressure $\sigma_{n}$ (used in the contact heat transfer model $h_{cont}$), and the contact area between the billet and the blocking dies.

### Appendix A.1. Mechanical Problem

The general governing equation for solid mechanics is

(A1) $$\rho \frac{\partial^{2}\vec{u}}{\partial t^{2}}-div\left(\sigma \right)=\vec{b_{0}}\;\mathrm{in}\;\Omega$$

where ρ(T) is the (temperature-dependent) material density, $\vec{u}$ is the displacement vector, σ is the stress tensor, and ${\vec{b}}_{0}$ is the body force vector (typically representing gravity, ${\vec{b}}_{0}=\rho \vec{g}$). However, for hot forging simulations the inertial term can be neglected.

To close the system in Equation (A1), appropriate constitutive models are introduced for each component. The billet is assumed to behave as a viscoplastic material, and its flow stress is modelled using the Hansel–Spittel formulation:

(A2) $$\sigma =A\cdot \exp\left(m_{1}T\right)\cdot \varepsilon^{m_{2}}\cdot \exp\left(\frac{m_{4}}{\varepsilon }\right)\cdot {\dot{\varepsilon }}^{m_{3}}$$

where σ is the flow stress, *T* the temperature, ε the plastic strain, and $\dot{\varepsilon }$ the plastic strain rate. The material constants used for the billet are A=1809.24, $m_{1}=-0.0029$, $m_{2}=-0.14227$, $m_{3}=0.14102$, and $m_{4}=-0.05764$. For the dies, an elasto-plastic behaviour is considered. Their flow stress is also described using the Hansel–Spittel law, with A=2745.26, $m_{1}=-0.0009$, and $m_{2}=0.09060$, while $m_{3}=m_{4}=0$. These models allow incorporating strain, strain rate, and temperature effects relevant to hot forging processes.

For this problem, the domain boundaries can be divided into three distinct zones, each associated with a specific boundary condition:

- **Fixed support (Dirichlet condition)**: The bottom surface of the lower die, which is in contact with the press base (or carriage), is assumed to be fully constrained. Displacement is blocked in all directions at this boundary:
  (A3) $$\vec{u}\left({\vec{x}}_{\mathrm{blocked}}\right)=\vec{0}\;\mathrm{on}\;\Gamma_{D_{1}}$$
- **Prescribed displacement**: The top surface of the upper die, which interfaces with the press hammer, is subjected to a prescribed downward motion to replicate the mechanical action of the press:
  (A4) $$\vec{u}\left({\vec{x}}_{\mathrm{press}}\right)={\vec{u}}_{\mathrm{press}}\;\mathrm{on}\;\Gamma_{D_{2}}$$
- **Free surface (Neumann condition)**: The remaining surfaces of both dies are considered free from external mechanical loads. On these boundaries, a zero-stress (traction-free) condition is applied:
  (A5) $$\sigma \cdot \vec{n}=\vec{0}\;\mathrm{on}\;\Gamma_{L}$$

When contact occurs between the punch and the die, a normal contact pressure $p_{N}$ appears on the contact surface:

(A6) $$\left(\sigma \cdot \vec{n}\right)\cdot \vec{n}=\sigma_{n},\;\mathrm{with}\;\sigma_{n}\leq 0$$

The gap function $g(\vec{u})$ determines whether contact is active:

(A7) $$g\left(\vec{u}\right)=\left(\vec{h}-\vec{u}\right)\cdot \vec{n}\geq 0$$

where $\vec{h}$ is the initial separation. Contact conditions are then defined by the Hertz–Signorini–Moreau conditions:

(A8) $$\left\{\begin{array}{c} g(\vec{u})\geq 0\\ \sigma_{n}\leq 0\\ g\left(\vec{u}\right)\sigma_{n}=0 \end{array}\right.\;\mathrm{on}\;\Gamma_{C}$$

If contact is active, two scenarios are possible:

- **Sticking**: no relative tangential motion.
- **Sliding**: tangential motion occurs.

Friction is modelled using Coulomb’s law, which defines the relationship between tangential and normal contact pressures:

(A9) $$\sigma_{T}+\mu \sigma_{n}\leq 0$$

where μ is the friction coefficient.

Conditions for friction are given by

(A10) $$\left\{\begin{array}{c} |u_{T}|\geq 0\\ \sigma_{T}\leq 0\\ |u_{T}|p_{T}=0 \end{array}\right.\;\mathrm{on}\;\Gamma_{C}$$

The combined stress vector on the contact boundary is then

(A11) $$\sigma \cdot \vec{n}=\sigma_{n}\vec{n}+\sigma_{T}\vec{t}$$

where $\vec{t}$ is the tangential direction at the contact point.

### Appendix A.2. Thermomechanical Coupling Strategy

The problem during the billet deformation requires implementation of the numerical coupling of the thermal and mechanical models, as each model influences the other:

- The material properties (elasticity, strength, and other mechanical properties) vary with temperature.
- Temperature gradients cause expansions and contractions, generating mechanical stresses.
- Mechanical processes (i.e., deformation and friction between the solids) generate the heat source *Q* that must be considered in the thermal problem (1).

FORGE NxT 3.2 resolves coupled problems by associating temperature-dependent mechanical properties at each node. The coupling process follows these steps:

1. Initialises the simulation with thermal and mechanical conditions.
2. Detects contact areas between components.
3. Computes mechanical properties based on the local temperature: E(T), ρ(T), ν(T), etc.
4. Solves the mechanical problem.
5. Calculates heat sources:
  - Internal heat *Q* from plastic deformation.
  - Surface heat flux $q_{\mathrm{cont}}$ due to contact and friction (see Equation (6)).
6. Solves the thermal problem, incorporating all defined boundary conditions.
7. Checks for convergence:
  - If not converged: update properties and repeat.
  - If converged: advance to the next time step.

This process repeats until the final simulation time is reached. Figure A1 shows the coupling algorithm scheme used by FORGE NxT 3.2.
